# The 3D-Printing-Accelerated Design for a Biodegradable Respirator from Tree Leaves (TRespirator)

**DOI:** 10.3390/polym14091681

**Published:** 2022-04-21

**Authors:** Ziao Wang, Yao Xu, Rulin Liu, Xi Zhu

**Affiliations:** School of Science and Engineering (SSE), Shenzhen Institute of Artificial Intelligence and Robotics for Society (AIRS), The Chinese University of Hong Kong, Shenzhen (CUHK-Shenzhen), Shenzhen 518172, China; ziaowang@link.cuhk.edu.cn (Z.W.); yaoxu@link.cuhk.edu.cn (Y.X.); rulinliu@link.cuhk.edu.cn (R.L.)

**Keywords:** biodegradable masks, high precision 3D printing, microplastic, mechanical properties, plastic pollution problem

## Abstract

The unpredictable coronavirus pandemic (COVID-19) has led to a sudden and massive demand for face masks, leading to severe plastic pollution. Here, we propose a method for manufacturing biodegradable masks using high-precision 3D printing technology, called “TRespirator”, mainly made of banana leaves and dental floss silk fibers. By adding plastic recycling waste appropriately, TRespirator can achieve similar protection and mechanical properties as N95 masks. In addition, microorganisms attracted during the degradation of plant fibers will accelerate the degradation of microplastics. This respirator provides a new idea for solving the global problem of plastic pollution of masks.

## 1. Introduction

The unpredictable and unprecedented coronavirus pandemic (COVID-19) has driven a sudden massive demand for face masks [1,2]. Recent studies estimated that nearly 5 billion face masks are required every day this year [3], which has caused environmental problems related to the waste of a large number of masks [4,5]. Randomly-discarded face masks have led to severe plastic pollution [6]. Most face masks are composed of two nonwoven fabric cloths with a middle layer made of melt-blowing cloths. Their main component is polypropylene (PP), the leading cause of plastic pollution because of its long degradation time in nature [7,8,9]. A general mask degrades slowly in soil, which would take approximately 450 years to decompose fully [10]. Even though there are popular biodegradable plastic products made of polylactic acid (PLA), its degradation process would also generate microplastic, which is not degradable, causing secondary pollution [11].

Microplastics (MPs) are formed during the degrading of face masks, and the MPs particles are usually only in the range of a few microns [12]. Now, MP has been found in places where surveys have been conducted worldwide [6]. Due to the slow degradation and mineralization rates of the most commonly used plastics, these tiny fragments far outweigh the larger, more visible fragments of plastic debris in the environment [9], which contains a potential risk to human health [13,14]. In the United States, MPs are estimated to be consumed through the food chain and inhalation pathways at 74,000–211,000 particles per year. MPs have also been detected in human feces [15]. To deal with the MPs pollution from the wasted face masks, some solutions have been proposed, such as replacing the raw material for masks [16], improving mask recycling efficiency, and using reusable masks [17]. However, most of these methods are economically unfavorable; thus, more efficient and lower-cost materials are urgent.

This work reports a plant fiber face mask called ‘TRespirator’ that is entirely biodegradable. It is mainly made of natural products, banana leaves, and floss silk. Only about 5% weight percent of the TRespirator is made of recycled plastic bottles waste as the adhesive of fibers, which would reduce raw materials cost to only about $2 for the fabrication of a thousand TRespirators. Micro-structure design and 3D printing were applied to ensure breathability and filtration efficiency performance with less plastic. We developed an accelerated degradation-test model to predict the natural degradation period of materials within 24 h. We optimized the performance and degradation character of 20 kinds of plant leaves. The plant-fiber-plastic system shortens the degradation time of the Trespirator because the plant fiber degradation would bring enzymes in the soil to the TRespirator system and make enzymes locate and bio-degrade MPs easier. The results show that the TRespirator is an excellent solution to severe plastic pollution by increasing the consumption of disposable masks.

## 2. Experimental Methods

### 2.1. Materials and Chemicals

The withered banana leaves and falling floss silk were collected from fallen-leaf piles. Polyethylene terephthalate (PET) was collected from waste plastic bottles. Hydrogen peroxide (H_2_O_2_, Synth, Maharashtra, India), Sodium hydroxide (NaOH, Synth, Maharashtra, India), Antimony (III) oxide (Sb_2_O_3_, 3A Chemicals, Nagpur, India), and ethylene glycol ((CH_2_OH)_2_, Carlo Erba, Val de Reuil, France) were used as received.

### 2.2. Preparation of Plant Fibers

To obtain fibers from leaves, leaf meat needed to be removed [18]. The banana leaves were immersed in deionized water (stirred at 80 ℃ for 1 h under stirring) and then washed in flowing water to remove impurities and soften the leaves. To isolate fibers in banana leaves, the leaves were heated in 200 mL of a 2 mol/L NaOH solution at 80 ℃ for 1 h under stirring. The mixture was cooled to room temperature, and filtered plant fibers were washed with deionized water until pH = 8, a nearly neutral pH. The cleaned fibers were dried at 80 ℃ in a vacuum until the weight no longer changed. Floss silk fibers were treated similarly to banana leaves to remove sugars and impurities.

### 2.3. Fabrication of TRespirator

The filter layer was made of plant fibers and the plasticizer, PET, from the waste of the plastic bottles. Antimony (III) oxide was used as the catalyst to accelerate the dissolution and adhesion of leaf fibers. Moreover, (CH_2_OH)_2_ was used as the solvent in the preparation process [19]. PET and banana leaf fibers, with fibers weight of 90%, were mixed under heating to 200 ℃ and stirred with the catalyst Sb_2_O_3_. After completely melting PET and mixing the mixture, ethylene glycol was excessively added to the system to ensure that the whole process took place in the solvent. Because ethylene glycol is volatile, there was always fog in the bottle. The heating ended until there was little fog, which showed that the solvent had almost evaporated. The products were cooled until room temperature and put into a 3D printer (Nanoscribe, Photonic Professional GT218, Karlsruhe, Germany) to obtain the TRespirator. The pre-designed print profile and desired size were inputted into the 3D printing setting system. (The desired size is similar to the general face mask; the pre-designed structure is shown in Section 3). The synthesized product was placed in the feed container of the printer under room temperature, and the unwanted parts were etched away by laser in an enclosed space inside the printer to generate the desired TRespirator.

More detailed methods, such as the blenching of fibers and product sterilization methods, are listed and wholly described in Supplementary S1: The reaction process of the treatment of banana leaf fibers and floss silk fibers and the fabrication of the TRespirator.

## 3. Results and Discussion

**Figure 1:**
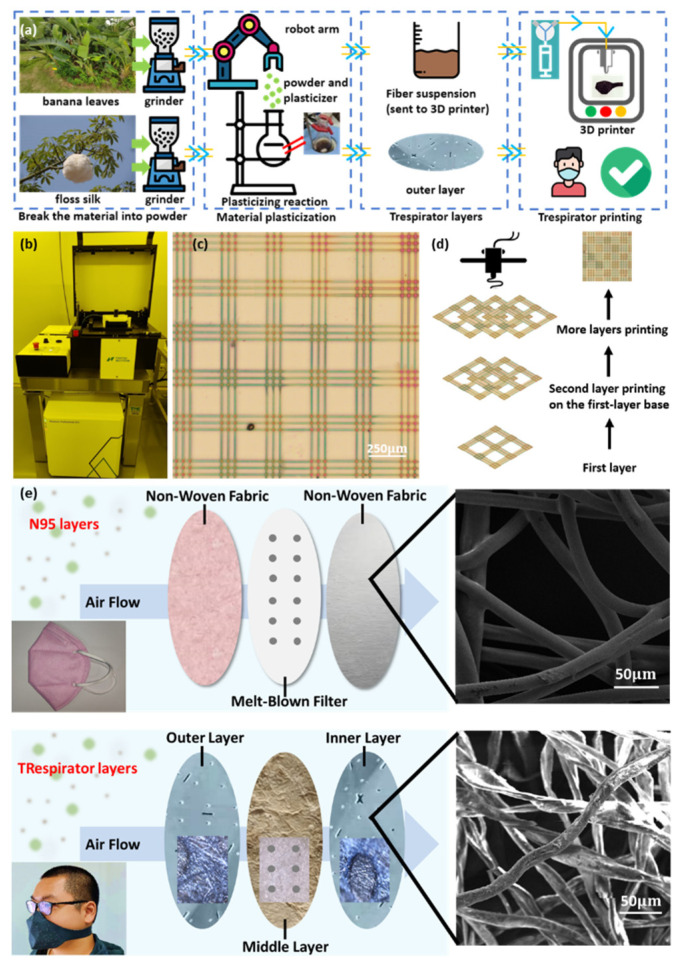
The synthesis and structure of the TRespirator and comparison to a N95 mask. (**a**) The reaction process of preparing banana leaf fibers and floss silk fibers, which are the components of the inner layer and two outer layers, respectively. After the plasticizing reaction, the fiber suspension of banana leaves was sent to the 3D printer to fabricate the middle layer. (**b**) The overall photo of the 3D printing machine, (**c**) the printed single layer of the middle layer of the TRespirator, (**d**) the schematic process diagram of multiple-layer printing, (**e**) the 3-layer-structure comparison between the N95 mask and the TRespirator, with two outer layers blocking big impurities, and the inner layer filtering small particles. Grey dots indicate the electrostatic electret sites.

The preparation of banana leaf fibers and floss silk fibers is shown in Figure 1a (Supplementary S1). Alkali treatment on the fibers was used to clean the impurities and hydrophilic groups on the surface of fibers, such as cellulose, lignin, etc. [20]. Two kinds of fibers are chemically treated and ground separately to synthesize different materials. The mask’s middle layers, made of melt-blown cloth, play the primary role in filtering impurities. To achieve high air permeability and filtration efficiency with less plastic add-in, we designed a fiber-crossing model and a high-precision 3D printer (Nanoscribe, Photonic Professional GT218, Karlsruhe, Germany) to control the shape of the TRespirator perfectly, as shown in Figure 1b. The GT2 prints 3D structure by layer-by-layer two-photon polymerization, which is the main reason for its high precision with the finest vertical resolution of 400 nm. Using galvo technology, we could also make the 3D printer achieve high-speed 3D microfabrication [21]. Figure 1c shows the microstructure of the optimized TRespirator, and the printing process of this structure is shown in Supplementary Video S1. Fibers cross each other to filter the impurities, with the spacing between the crossing part providing excellent air permeability. Meanwhile, multiple layers would avoid impurities across the spacing part, with the diameter of pores about 50 μm. As shown in Figure 1d, the crossing of part A in the second layer would cover the spacing of part B in the lower layer. Every layer staggers each other, blocking PM effectively with minimal loss of air permeability. Red points on the crossing point were printed by PET, which could also provide the mechanical intensity and ensure the durability of the TRespirator. Therefore, only a tiny proportion of PET plastic is required to achieve suitable mechanism strength, a particulate matter (PM) removal effect, and breathability.

Referring to the layered structure of N95 masks, as shown in Figure 1e, two outer layers of masks are used to block large-size impurities with the same material. Therefore, a thick fiber that would adsorb the big particle would be chosen as the raw material of the outer layer. The floss silk contains a fiber diameter of 20 μm, which is similar to the diameter of fibers in the outer layers of N95 masks. Meanwhile, the anti-bacterial effect of floss silk, which is the main reason for using floss silk in dentistry, would also make the TRespirator more protective. Because floss silk is used widely in daily cloth, the comfort of the layer next to users’ faces could also be ensured. Therefore, floss silk was chosen as the raw material for the two outer layers. The banana leaf and floss silk layers would perform well against air pollution and pandemics, such as COVID-19.

To optimize the raw material choice to obtain excellent mask performance, we built a mathematical model of the scoring system to evaluate the performance of the mask. The performance of the masks is related to both their permeability and filtration efficiency. The permeability was calculated from Darcy’s Law [22]. The flux equals:

(1) $$U=-c\frac{R^{2}}{8}\frac{\alpha }{\tau }\frac{1}{\eta }\nabla p$$

where c is an empirical geometrical factor, R is the radius of the holes, α is the porosity, τ is the tortuosity and equals 1 here, η is the viscosity, and p is the air pressure. It is supposed that the mask is made of vertical and horizontal fibers with a distance 2R and a diameter $d_{f}$. The radius can be expressed in its porosity since the area of the square holes can be computed. Since the flux is conserved before and after the mask, the pressure should be proportional to the area of the air channels. We can find:

(2) $$U=-c\frac{R^{2}}{8}\frac{\alpha }{\tau }\frac{p_{0}}{\eta }\frac{{\left(R-d_{f}/2\right)}^{2}-R^{2}}{d_{f}}$$

With $p_{0}$ being the atmospheric pressure, which can be solved from the porosity α by

(3) $$\alpha =\frac{R^{2}-{\left(R-d_{f}\right)}^{2}}{R^{2}}\Rightarrow R=\frac{d_{f}\left(1-\sqrt{1-\alpha }\right)}{\alpha }$$

As for the filter efficiency, it is approximated as [23]

(4) $$F\propto \alpha \left(1-e^{-\left(\alpha -1\right)}\right)$$

Since it must be low in filter efficiency on both ends, the final performance index Q is

(5) $$Q=UF\propto -c\frac{R^{2}}{8}\frac{\alpha }{\tau }\frac{p_{0}}{\eta }\frac{{\left(R-d_{f}/2\right)}^{2}-R^{2}}{d_{f}}\left(1-e^{-\left(\alpha -1\right)}\right)$$

**Figure 2:**
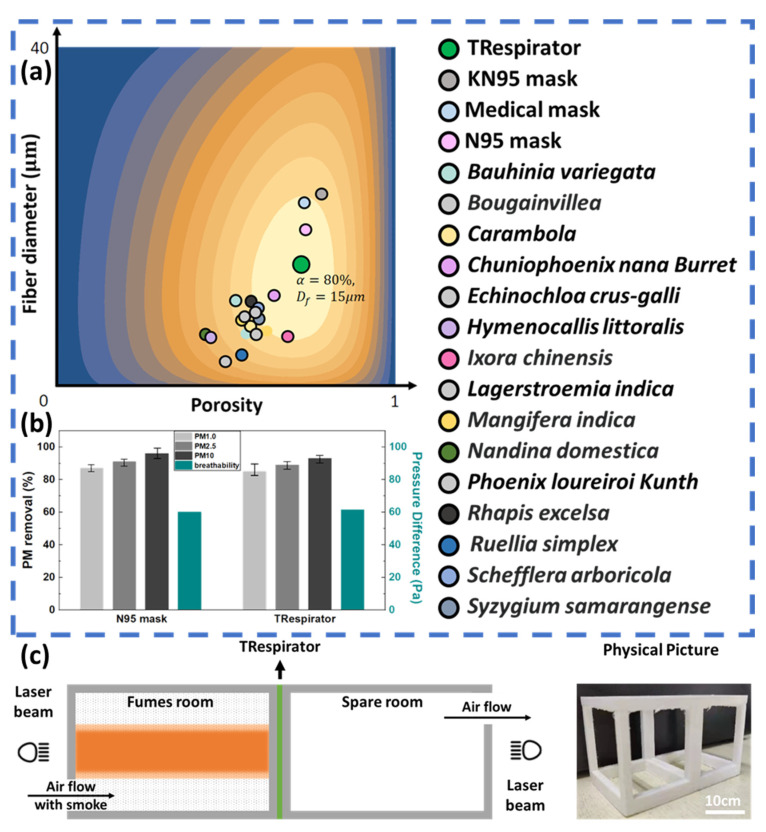
The experimental data about the performance examination of TRespirator (**a**) The heat map of the mask performance index Q, where the gray, light blue, and pink points indicate the corresponding coordinate position of KN95 mask, medical-surgical mask, and N95 mask from up to down, with the green one corresponding to the coordinate of 95% TRespirator, with remaining-colored points matching the other investigating plants listed on the right side. (**b**) PM removal efficiency (PM 1.0 in light grey, PM2.5 in grey, and PM 10 in black) and pressure difference (in blue) of TRespirator compared to those of N95 mask, with the error bar the standard deviation of all testing results, and (**c**) visual smoke adsorption test equipment of TRespirator.

The numerical result of this equation is shown in Figure 2a, where it can be observed that an optimal mask is found with a diameter of ~15 μm and porosity of ~80%. The green dot denotes the TRespirator, and the gray, light blue, and pink ones denote the KN95 mask, medical-surgical mask, and N95 mask from up to down, respectively [24]. To find the best raw material for TRespirator, 15 kinds of plants were investigated, and their properties of fiber diameter and porosity are shown as colored dots in the heat map (Supplementary Table S3). Results show that fibers with a larger diameter are more suitable to be the raw material of TRespirator.

We conducted tests for the TRespirator’s primary function, being breathable and protective. The sample’s air pressure difference (ΔP) is an essential indicator of filter performance [23]. Breathing through a filter with a high-pressure drop is uncomfortable for the user. Figure 2b shows the experimental data of the air pressure drop rate on the TRespirator and N95 medical masks. The pressure drops across TRespirator, and N95 medical masks were measured as around 61.4 Pa and 60 Pa, with air passing through the masks at velocities of 1.0 m/s, close to the human exhalation rate (about 1.3 m/s during breathing) [25]. The TRespirator displayed a higher pressure drop than the N95 mask at a similar thickness because the plant fiber layers have small pores.

The PM removal efficiency of the N95 medical mask and TRespirator was investigated without an electrostatic charge at the target air velocity of 1.0 m s^−1^. The results are shown in Figure 2b. The PM removal efficiency was proportional to the thickness and PM size generally. The removal efficiency of the TRespirator is near to that of the N95 mask. The N95 mask exhibited removal efficiency of 87%, 91%, and 96% for PM1.0, PM2.5, and PM10, respectively, while the corresponding values for the TRespirator are 81%, 85%, and 92%. In the absence of an electrostatic charge, physical sieving is limited in its ability to simultaneously achieve the desired pressure drop and removal efficiency because of their trade-off relationship. Further, we applied the electrostatic technique to our product and conducted the filtration efficiency test. The filtration efficiency of the TRespirator improved to 85%, 89%, and 93%, which is close to that of the N95 mask. The adsorption effect of plant fiber and the staggered structure between layers can also help improve the filtration efficiency of the TRespirator close to that of N95 masks.

The effective PM barrier performance of the TRespirator is visually shown in Figure 2c. Burning cigarettes can produce severe PM. The filter between the PM source and another empty box was visually confirmed to prevent the generated smoke from entering the empty box through the Tyndall effect. The surface layer made of floss silk fibers blocks the relatively large PM, and the middle layer, composed of banana leaf fibers, adsorbs relatively small PM.

For the increasing demand and consumption of masks, recent works proposed multiple-use masks as the solution because they could effectively decrease masks’ depletion and reduce the masks recycling cost. However, most face masks would suffer performance losses after being used in ambient humidity, and bacteria filtered by masks would harm users again. The main reason for the choice of multiple-use masks is that these masks could not be biodegraded efficiently; of note, the TRespirator is biodegradable. We spent 8 h, which is nearly the maximum mask-wearing time in one day, testing the hydrophobicity of TRespirator and commercial N95 mask filters. The N95 mask filter, whose removal efficiency for PM1.0 is most affected by electrostatics, declined by more than 8%, which could be considered a significant performance loss for masks. In contrast, the TRespirator gradually loses performance and we cannot use the mask after about 20 h. Used TRespirators could be buried and biodegraded, which would lead to no pollution, reduce the recycling cost, and avoid the potential harm of used masks.

**Figure 3:**
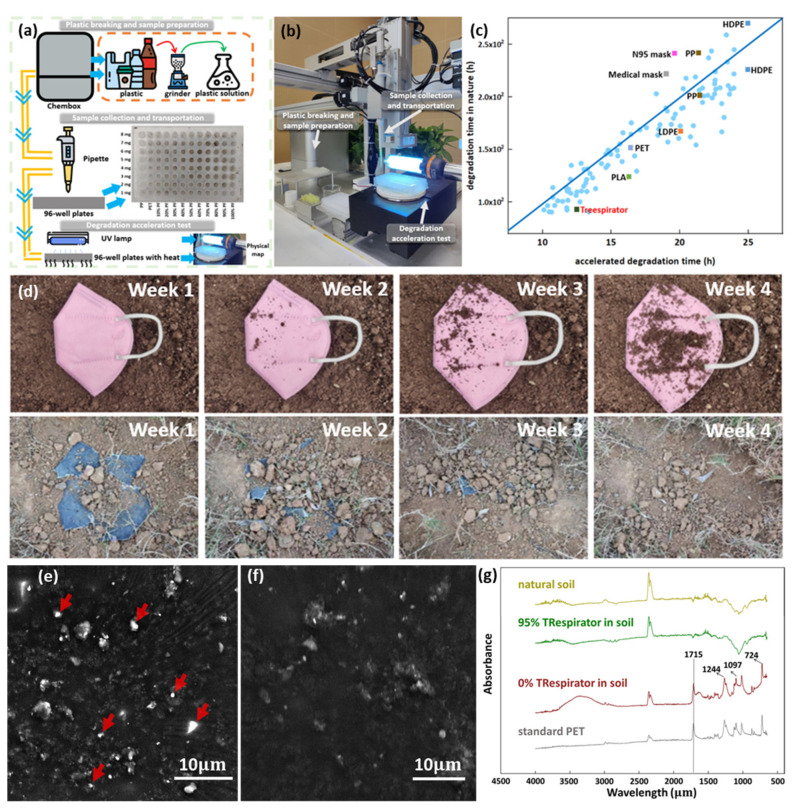
The degradation acceleration test and degradation performance re-check (**a**) the schematic diagram of the degradation acceleration test process. In the 96-well plate, the *x*-axis distributes 12 kinds of samples, which are PP sample, PET sample, plant-PET mixture samples from 10% plant fibers (PF) weight ratio to 100% weight ratio, and the *y*-axis is eight samples with different weights from 1 mg to 8 mg, (**b**) the physical map of the automatic degradation acceleration testing machine, (**c**) the image of the experimental data shown as light blue points, and the linear relationship between the degradation periods under acceleration and in nature, (**d**) the degradation process of a N95 mask and TRespirator from week 1 to week 4, (**e**,**f**) the SEM images of the soil in which the degradation test of the membrane without plant fibers and plant fiber membrane takes place, respectively, where the highlights are micro and nano plastics in different size (**g**) the FTIR test of standard PET material, the soil after the degradation of the plastic membrane, the soil after TRespirator degradation, and natural soil sample; 95% TRespirator means a TRespirator sample with fibers 95 weight percent, and 0% TRespirator means a TRespirator with no fibers.

The degradation period is the crucial parameter for the degradability of materials. However, it is inefficient and high-cost to examine the degradation time of every material, some of which would take hundreds of years to decompose. Therefore, we use a high-throughput degradation method to investigate the relationship between the degradation period under a specific environment capable of rapid degradation and the period in nature. Two 96-well plates with the same arrangement with different material types and amounts were prepared and tested under an accelerated degradation environment [26] and natural degradation, respectively, with the reaction process shown in Figure 3a and the robot for the reaction shown in Figure 3b (Supplementary S2). The time-dependent mass decay under a typical environment is exponential as $e^{-t/t_{0}}$, while under ultraviolet heating, it becomes $e^{-t/t_{1}}$, where $t_{1}\ll t_{0}$ [27]. Thus, the time scale of the two should be linear, i.e., $t_{env}=t_{ult}\times t_{0}/t_{1}$. The factor $t_{0}/t_{1}$, though the two-time constant is expected to be different, it would be similar for the materials as it refers to the environmental change from natural soil to ultraviolet heating. Thus, the factor would be obtained with a linear regressor with data from high throughput experiments.

Figure 3c compares the decomposition in the high throughput experiments and by rotting in the soil, with several highlights of the degradation data of the TRespirator, N95 mask, medical mask, and references [28,29,30]. For each sample pair of the same material decayed to the same residual percentage, the time taken in both conditions was plotted as the horizontal and vertical coordinate. It can be observed that the time lengths of the two-scale show linear scaling, which agrees with the linearity of the factor $t_{0}/t_{1}$ above. The ultraviolet heating was found to accelerate the decomposition by 10 times. Therefore, with the model, the degradation time of material could be predicted in a short period of the degradation acceleration experiment, which assists in determining banana leaves and floss silk as the raw material of the TRespirator because of their excellent degradation performance in the acceleration test.

Figure 3d shows the degradation performance of the N95 mask and TRespirator in soil, respectively. Two samples were dug out at regular intervals for photographing and recording. The TRespirator began to be biodegraded in a week. Within 4 weeks, TRespirator was decomposed in the compost soil at room temperature and sunlight, and after week 2, the degradation effect became apparent by the aggregation of microbial. In contrast, the N95 mask had nearly no change in 4 weeks in soil. After the degradation experiment, the soil was sampled and characterized to test whether TRespirator was degraded. The experimental soil of 0% TRespirator and 95% TRespirator was collected and characterized by SEM images and FTIR investigation to check if PET nanoparticles were in the samples.

Figure 3e,f shows the SEM images of the soil of 0% TRespirator and 95% TRespirator. The soil sample after the degradation of 0% TRespirator contains MP particles in different sizes, from 100 nm to about 1.7 μm (Supplementary Figure S16). In contrast, the other image (Figure 3f) only shows some soil particles and plant fibers remaining. FTIR investigations of pure PET, 0% TRespirator in soil, 95% TRespirator in soil, and natural soil samples are shown in Figure 3g. Pure PET and natural soil were investigated as control groups. Results show that only the soil sample after 0% TRespirator degradation contained PET. Still, there are two similar images of the 95% TRespirator in soil and natural soil, which mirrors a similar conclusion from SEM images.

**Figure 4:**
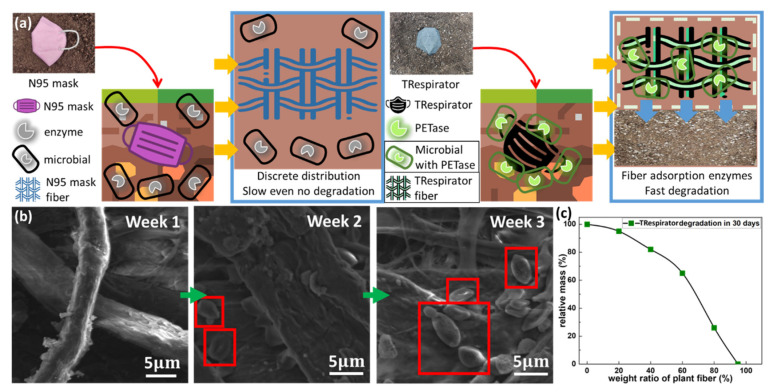
The degradation mechanism of the plant-fiber-plastic system in the TRespirator (**a**) The degradation mechanism of an N95 mask and TRespirator, (**b**) SEM images of degrading TRespirator samples from week 1 to week 3, (**c**) image about the degradation effect in 30 days with a different weight ratio of plant fibers.

MP pollution is being intensely focused on because it cannot be degraded rapidly and collected effectively. It is undegradable because it is plastic, but its tiny size gives more difficulty to its degradation in nature [31]. Under a critical size, the deformation of the smaller fragment is plastically rather than cracking, in which there would be tremendous input energy wasted [32]. Therefore, there is little effective energy to break the covalent bonds between monomers in a small polymer fragment system. Figure 4a shows the degradation diagram of the N95 mask. Macro plastic cannot be degraded because of its strong covalent bond between plastic monomers. MP also cannot be degraded because of its surface activity in its tiny size. Even if there are enzyme bio-based reactants in the soil, they cannot contact MP effectively, making it hard to degrade MP naturally.

However, the addition of plant fiber makes the degradation of MP possible. Recent studies have confirmed that MPs can be depolymerized and degraded by confined enzymes [33]. Besides, some carboxylesterases (Enzyme Commission (EC) number 3.1.1.1), lipases (EC 3.1.13), and cutinases from various microbes can hydrolyze PET [34]. Cutin on the surface of floss silk can attract bacteria that contain cutinases (EC 3.1.1.74) [34,35]. PET, which was used in the TRespirator as the adhesive, was naturally and efficiently degraded by IsPETase, a kind of cutinase found in 2016 [35,36]. In a PET-rich environment, the flexibility of the substrate-binding pocket in IsPETase increases, which changes the preference of enzyme substrates for large substrates, such as PET. Other similar sequences with IsPETase can also improve the hydrolytic activity of PET through this soil pretreatment [36]. Therefore, as shown in Figure 4a, in the degradation of TRespirator, floss silk fibers bring kinds of microbes with cutinases to this system, leading to the acceleration of the degradation of MP. In contrast, without plants attracting microbes to the mask, enzymes cannot contact plastics, which leads to degradation being unable to occur effectively [37]. Therefore, in TRespirator, the mixture of leaf fibers and plastic leads to a good degradation effect in nature.

Figure 4b shows the microbial aggregation effect on the TRespirator. The TRespirator samples in the beginning and after two-week degradation were collected and investigated by the microscope. Only fibers at the start and microbial consortium were aggregated around the fibers after degrading for two weeks. To optimize the proportion of plants in the TRespirator, we test the degradation performance of six different TRespirator samples with the weight ratio of 0%, 20%, 40%, 60%, 80%, and 95% of the plant fibers. Figure 4c shows the relative mass of TRespirator samples after a 30-day degradation. It shows that TRespirator made of 95% weight percent of plant fiber reached nearly total plastic degradation.

## 4. Conclusions

In summary, a biodegradable high-efficiency mask TRespirator was prepared using banana leaf fibers, floss silk, and PET waste. Three-dimensional printing is used to control the microstructure of the middle layer of the TRespirator. Because IsPETase is a kind of cutinase that can degrade silk fibers, PET degradation can be achieved by attracting the microbes in the soil containing IsPETase to the TRespirator system by the floss silk fibers. Unlike commercial disposable masks, the developed filter is disposable to avoid possible secondary harm using the masks. It completely biodegrades within a month in composted soil. In addition, the TRespirator has a PM removal rate of more than 80% of PM greater than 1 µm in size and 90% of that greater than 10 µm and delivered an acceptable air pressure drop of 61.4 Pa. These results indicate that we have successfully developed an environmentally friendly mask that provides a comfortable breathing environment for the user with little reduction in performance. This mask is expected to be a practical solution to nano plastic pollution and protect humans against COVID-19. Meanwhile, products that directly add natural products to existing industrial materials will have broader application scenarios in the future.

## 5. Methods

Fabrication of the TRespirator. Methods for the preparation of fibers and the fabrication of the TRespirator are listed in Supplementary S1 [38,39].

Characterization and performance test. The characterization and performance tests of the samples are listed in Supplementary S2 [40,41].

The chemical processes in the synthesis, the degradation, and the anti-bacterial property of the TRespirator are listed in Supplementary S3 [42,43,44,45].

## Data Availability

The data presented in this study are available on request from the corresponding author.

## Supplementary Materials

The following supporting information can be downloaded at: https://www.mdpi.com/article/10.3390/polym14091681/s1, Figure S1: The components of commercial masks; Figure S2: The properties of commercial masks; Figure S3: The appearance and the SEM image of Roystonea regia leaf; Figure S4: The appearance and the SEM image of pea leaf; Figure S5: The appearance and the SEM image of Bougainvillea glabra; Figure S6: The appearance and the SEM image of Roystonea regia leaf; Figure S7: The appearance and the SEM image of Hymenocallis littoralis leaf; Figure S8: The appearance and the SEM image of bamboo palm leaf; Figure S9: The appearance and the SEM image of Fissistigma oldhamii leaf; Figure S10: The appearance and the SEM image of Semarang rose-apple leaf; Figure S11: The appearance and the SEM image of fishtail palm leaf; Figure S12: The appearance and the SEM image of mango leaf; Figure S13: The appearance and the SEM image of Lagerstroemia indica leaf; Figure S14: The appearance and the SEM image of Chinese Ixora leaf; Figure S15: The appearance and the SEM image of Britton’s wild petunia leaf; Figure S16: The SEM image and size distribution of microplastic in the soil after deg radation of the medical-surgical mask; Figure S17: The relationship between (a) the number of 3D printing layers and air permeability (b) the number of 3D printing layers and filtration efficiency of the TRespirator; Figure S18: Characterization and performance test machines; Table S1: The cost for an N95 mask; Table S2: the diameter, pore size, with mean and maximum pore size respectively, and porosity of the non-woven medical surgical mask, hot air cotton layer of the KN95 mask, and hot air cotton layer of N95 mask; Table S3: the fiber diameter, fiber length, and porosity of 21 kinds of plants; Video S1: 3D-printing process on the mixed photoresist.
