# Supplementary material for: The 3D-Printing-Accelerated Design for a Biodegradable Respirator from Tree Leaves (TRespirator)
Source: Polymers (Basel). 2022 Apr 21;14(9):1681. doi: 10.3390/polym14091681 (PMC9103532; doi:10.3390/polym14091681)
Supplement: Supplementary file 1 [file polymers-14-01681-s001.zip › polymers-1679289-SP1 updated.pdf]

Supplementary Information for

The 3D-Printing-Accelerated Design for A Biodegradable Respirator from Tree Leaves (TRespirator)

*Ziao Wang, Yao Xu, Rulin Liu, Xi Zhu**

School of Science and Engineering (SSE)

Shenzhen Institute of Artificial Intelligence and Robotics for Society (AIRS),

The Chinese University of Hong Kong, Shenzhen (CUHK-Shenzhen),

Shenzhen, Guangdong, P. R. China. 518172

Email: zhuxi@cuhk.edu.cn

This supplementary information file includes:

- Supplementary Notes S1 –S3
- Supplementary Figures S1 – S18
- Supplementary Tables S1 –S 3
- Supplementary Video S1
- Supplementary References

Supplementary Notes

S1: The reaction process of the treatment of banana leaf fibers and floss silk fibers and the fabrication of TRespirator.

Materials and chemicals The withered banana leaves and falling floss silk were purchased from the department to collect fallen leaves. Polyethylene terephthalate (PET) was collected from the waste of the plastic bottles. Hydrogen peroxide (H_2O_2, General Reagent), Sodium hydroxide (NaOH, General Reagent), Antimony (III) oxide (Sb_2O_3, 3A Chemicals), and ethylene glycol ($(CH_2OH)_2$, General Reagent) were used as received.

Preparation of plant fibers To obtain fibers from leaves, leaf meat needed to be removed[18]. The banana leaves were immersed in deionized water (stirred at 80°C for 1 h under stirring) and then washed in running water to remove impurities and soften leaves. To isolate fibers in banana leaves, the leaves were heated in 200 mL of a 2mol/L NaOH solution at 80°C for 1h under stirring. Then the mixture was cooled to room temperature, and filtered plant fibers were washed with deionized water until pH = 8, a nearly neutral pH. The cleaned fibers were dried at 80°C in a vacuum until the weight no longer changed. Floss silk fibers were treated similarly to banana leaves to remove sugars and impurities.

Bleaching of fibers To remove the pigment in the plant fibers from fibers and residual lignin and hemicelluloses in leaves, two kinds of fibers were treated with 30% H_2O_2 at 40°C for 1h under stirring. Then, the mixture was cooled at room temperature, filtered, and then dried at 80°C under vacuum until constant mass[38].

Fabrication of TRespirator The filter layer was made of plant fibers and the plasticizer, PET, from the waste of the plastic bottles. Antimony (III) oxide was used as the catalyst to accelerate the dissolution and adhesion of leaf fibers. And ethylene glycol was used as the solvent in the preparation process[19]. PET and banana leaf fibers, with fibers

weight 90%, were mixed under heating to 200 ℃ and stirring with the catalyst Sb_2O_3. After completely melting PET and mixing the mixture, ethylene glycol was excessively added to the system to ensure that the whole process took place in the solvent. Because ethylene glycol is volatile, there was always fog in the bottle. Heating ended until there was little fog out, which showed that the solvent almost evaporated. The products were cooled until room temperature and put into a 3D printer to get TRespirator.

Product sterilization method The TRespirator was sterilized by ethylene oxide (C_2H_4O, EO)[39]. Send the product to a special ethylene oxide sterilizer. Pour ethylene oxide gas into the container until the concentration reaches 800 mg/L～1000 mg/L. Warm up to 55°C～60°C, keep humidity between 60%～80%, and sterilize each batch of products for 6 hours.

S2: The characterization and the performance test of the TRespirator, masks, and plants.

Characterization The morphology of the TRespirator, masks, and plants were examined under scanning electron microscopy using a MAIA3-TESCAN microscope. The FTIR spectrometer examined the FTIR spectrum in the model of FTIR 850-Guangdong Sci&Tech. The pure PET microparticles, the soil after the plastic mask degradation, the soil after the TRespirator degradation, and the natural soil samples were prepared for the test.

Degradation performance test. A 96-well plate was used to collect samples. Different materials arranged the horizontal direction: PP, PET, and samples with different fiber weight ratios from 10% to 100%. The vertical direction was arranged by increasing sample weight from 0.5 mg to 4 mg. Two 96-well plates were prepared for the degradation acceleration and natural degradation tests, respectively. Samples were treated by acceleration degradation (70℃, 85%RH, and UV lighting) and natural degradation respectively and regularly monitored to test the degradability. Two mask

samples, a medical-surgical mask and the TRespirator, were put into the soil under solar light. The degradation processes of the two masks were recorded by taking out and taking photos every week for observation.

Air permeability test. The air permeability of the TRespirator was tested by an air permeability tester whose model is SG461-III [40]. TRespirator was blown with a continuous gas flow from down to up with 1 m/s. Collect the pressure difference data between two ends of the mask according to the pressure under and upon the mask measured by the instrument, and get the pressure drop rate and gas passing efficiency of the mask by the air pressure on the lower side divided by that on the upper side.

PM removal test. The PM removal was tested by a PM removal machine from Germany Eagle Technology Inc [41]. After the two kinds of masks are fixed with clips, the device generates airflow with particles to pass through the mask for testing. The Tyndall effect judged visual smoke isolation tests. A transparent box was separated by a panel with a hole to fix the TRespirator. A cigarette was burning in one-half of the box to generate enough smoke. Airflow from the smoking box to the other box was applied to ensure all the smoke was blown through TRespirator for the PM removal efficiency test. Meanwhile, two laser pointers were shooting to two boxes simultaneously to detect the Tyndall effect.

Moisture adsorption test. The moisture adsorption test took to examine how much the performance of the TRespirator was reduced and if TRespirator could reuse, which was measured as follows. Samples dried at 80 ℃ for 2 h were conditioned in airtight containers with a saturated calcium chloride solution to obtain a controlled environment with 55% RH. TRespirator after wetting was used to take the PM removal test and air permeability performance test again and get another set of data.

S3: Chemistry process

Treatment of lignin Lignin ($C_{18}H_{13}N_3Na_2O_8S_2$) is a complex organic polymer that

plays an important role in the structure of plant cell walls. However, considering the application in masks, the raw materials of masks need to be comfortable enough for the wearer, so the fibers need to have better softness. Still, lignin will keep the fibers hard, so we need alkali treatment on plants to remove the lignin from the fibers. Since lignin is soluble in water, the plant fiber is water-cleaned to remove some lignin from the surface. Then the alkali treatment is used on these fibers, with the experiment process described in **Supplementary S1 'Preparation of plant fibers'** section. Fiber will be processed further, and the processed lignin will be mixed with the photoresist required for 3D printing. They are mixed and heated to 50℃ for 1 h to mix well and then cool down to room temperature to prepare the raw material for 3D printing.

The degradation process of the TRespirator The degradation process of our samples in the high-throughout experiment is similar to the process and principle of the degradation and degradation of the leaves themselves.[42] The dead leaves fall from the trees, and the herb plants bear seeds and fall to the ground. These form a layer of rubbish on the surface of the soil. The volume of the cushion can be quite large. Bacterial hyphae will soon invade the rubbish. The mycelium draws nutrients from the garbage. This allows bacteria to grow and spread as they break down the structure of dead plant material. In our microstructural design, we designed to expose the plant fiber part at the endpoint so that when the TRespirator is buried and decomposed, bacteria can find a suitable site to start decomposition and carry out a biodegradation process similar to that of fallen leaves.

Anti-bacterial property The anti-bacterial properties of the floss silk and its attraction to microbials are realized in different environments. When we use TRespirator to block PM, the anti-bacterial property of floss silk will help the mask avoid viruses harming humankind[43]. However, the attraction to microbials during the biodegradation in the soil is essentially caused by the selective relationship between the enzyme and the target. And this attraction effect is manifested in that because the floss silk in the mask contains cutin, it will be selected by microbials containing specific enzymes during the

degradation process in the soil, rather than a process that actively adsorbs microbials everywhere. Therefore, TRespirator is safe, and the virus will only be isolated.

Meanwhile, silk's anti-bacterial property is mainly used to reduce *Staphylococcus aureus* colonization of the skin, which is beneficial in our product[44]. Therefore, microbials in the soil (*Actinomycete*, *Aerobacter*, and *Bacteroide,* for example[45]) would not be affected by the anti-bacteria performance of the floss silk, including the bacterias that contain *IsPETase* are not the target bacterias, and the biodegradation process can be normally addressed.

Storage and shelf life Because the TRespirator would be pre-treated, such as sterilization as mentioned above, and packaging protection, the product would not be polluted by bacteria in the air. Therefore, its shelf life would be much longer than its biodegradation period. After a test to expose the product completely to the outdoors, it spends 3 months beginning to appear performance loss.

S4 Mass production plan

Although the robotic aspect of this manufacturing may be scalable, 2-photon polymerization 3D printing may be more difficult to scale to the number/speed required to manufacture a global mask supply. To meet the worldwide demand for masks, a solution that can be mass-produced needs to be proposed. Here we can achieve mass production by combining the advantages of conventional-sized 3D printers and electrospinning. 3D printing achieves precise printing by pre-designing the graphic structure, which is why we use 3D printers. Meanwhile, electrospinning can achieve fiber diameter control in a small range. Therefore, we can first use electrospinning technology to control the diameter of the raw material within the diameter we need and then prepare the raw material for printing in a 3D printer to precisely control the microstructures we need.

Supplementary Figures

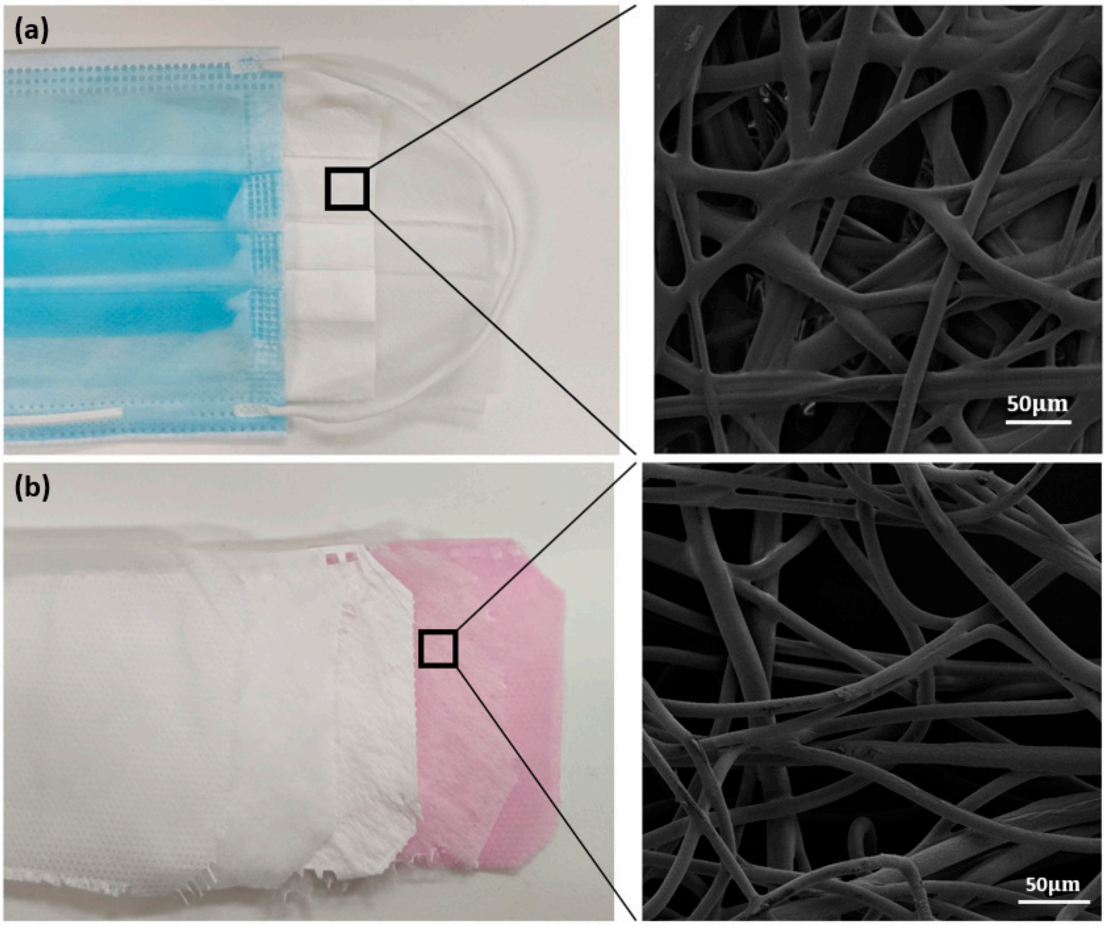

Supplementary Figure S1 The components of commercial masks (a) The components of the medical-surgical mask, with two outer layers of non-woven cloth and a middle layer of melt-blowing cloth, and the SEM image of the melt-blowing cloth in 50 μm, and **(b)** the components of the N95 mask, with two outer layers of non-woven cloth and a middle layer of melt-blowing cloth and two middle layers of hot air cotton besides the melt-blowing cloth, and the SEM image of the hot air cotton in 50 μm.

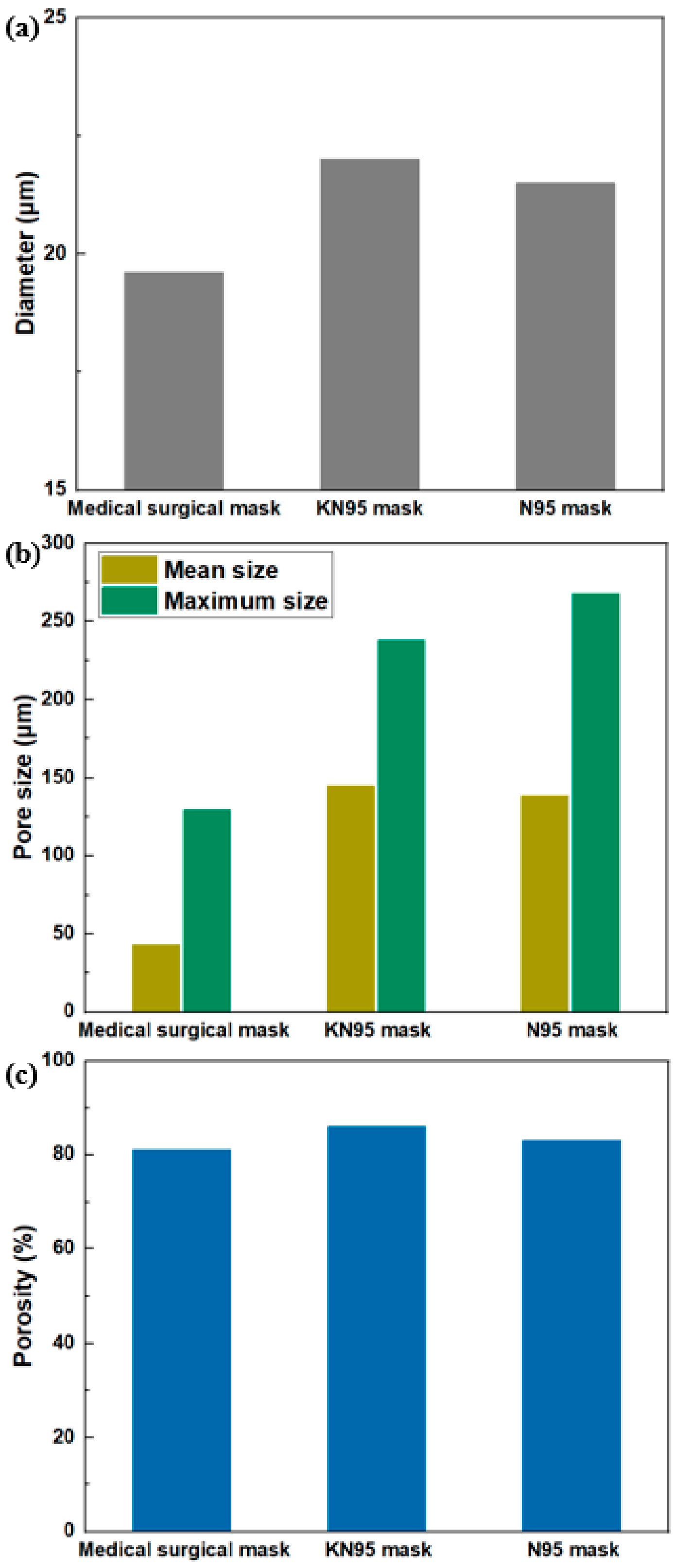

Supplementary Figure S2 The properties of commercial masks (a) the plastic fiber diameters, **(b)** the pore size, with mean size and maximum size respectively, and **(c)** the porosity of the medical-surgical mask, the KN95 mask, and an N95 mask.

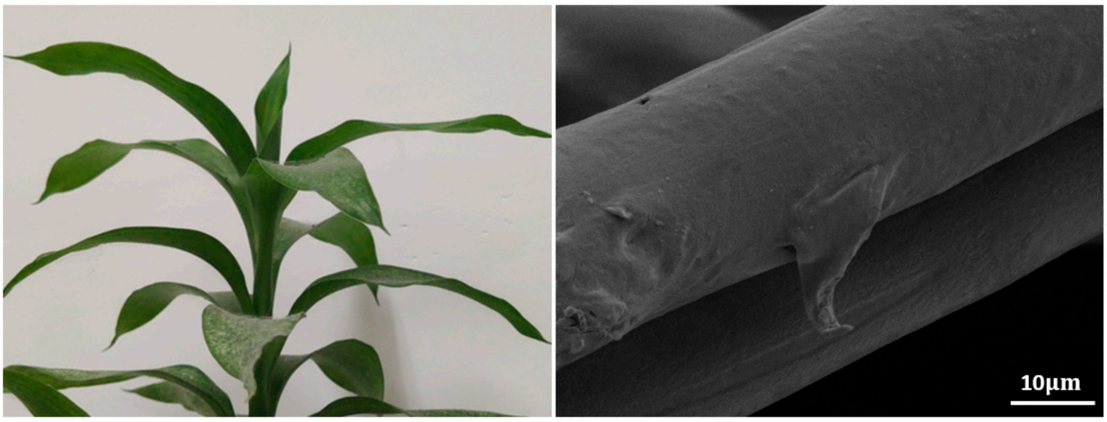

Supplementary Figure S3 The appearance and the SEM image of Roystonea regia leaf in 10μm.

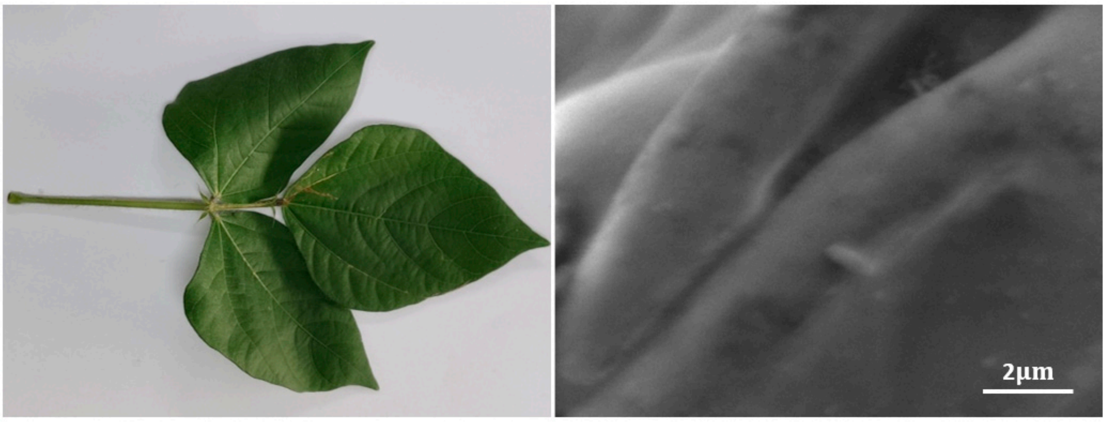

Supplementary Figure S4 The appearance and the SEM image of pea leaf in 2μm

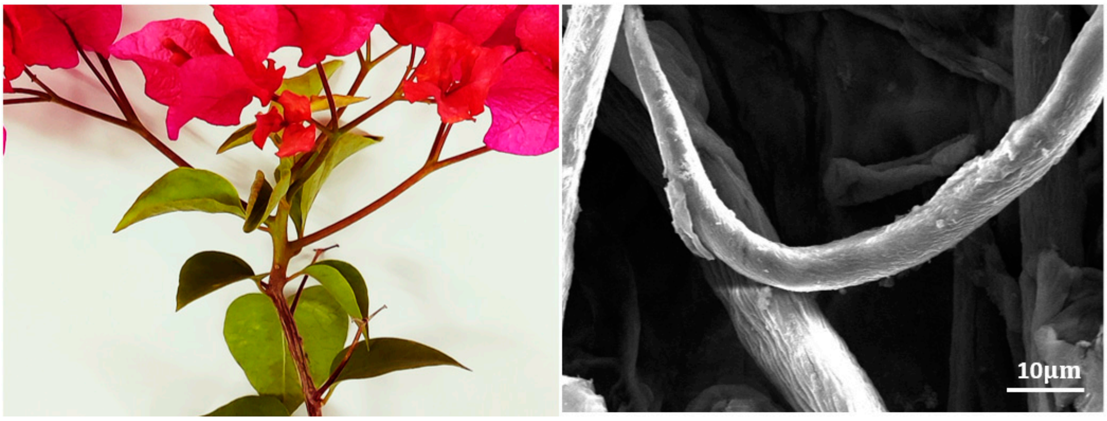

Supplementary Figure S5 The appearance and the SEM image of Bougainvillea glabra leaf in 10μm

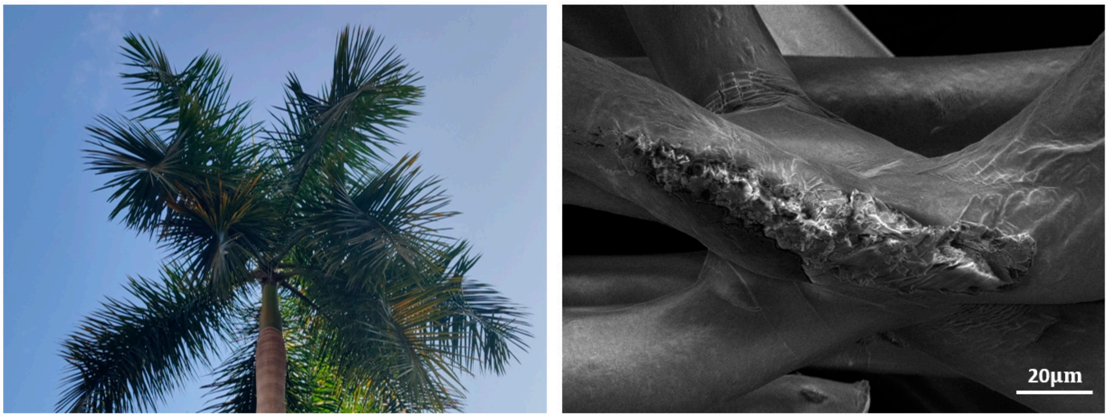

Supplementary Figure S6 The appearance and the SEM image of Roystonea regia leaf in 20μm

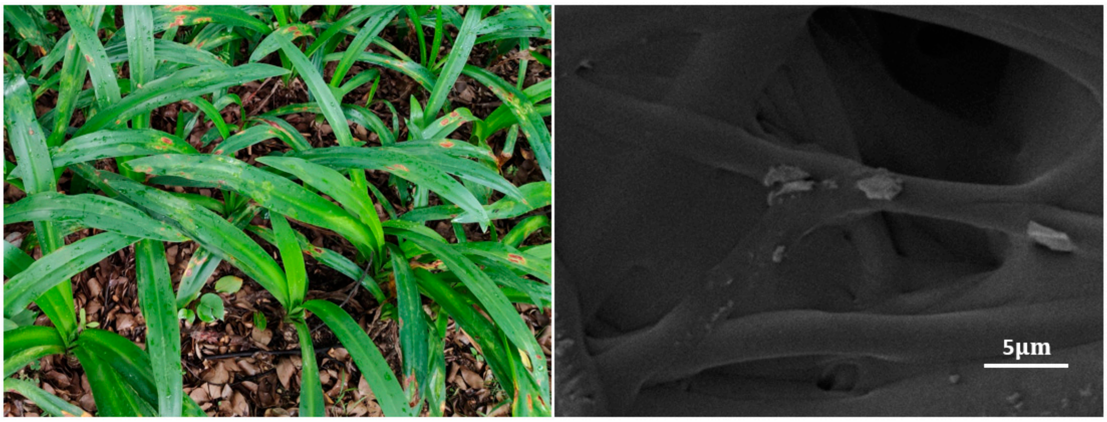

Supplementary Figure S7 The appearance and the SEM image of *Hymenocallis littoralis* leaf in 5μm

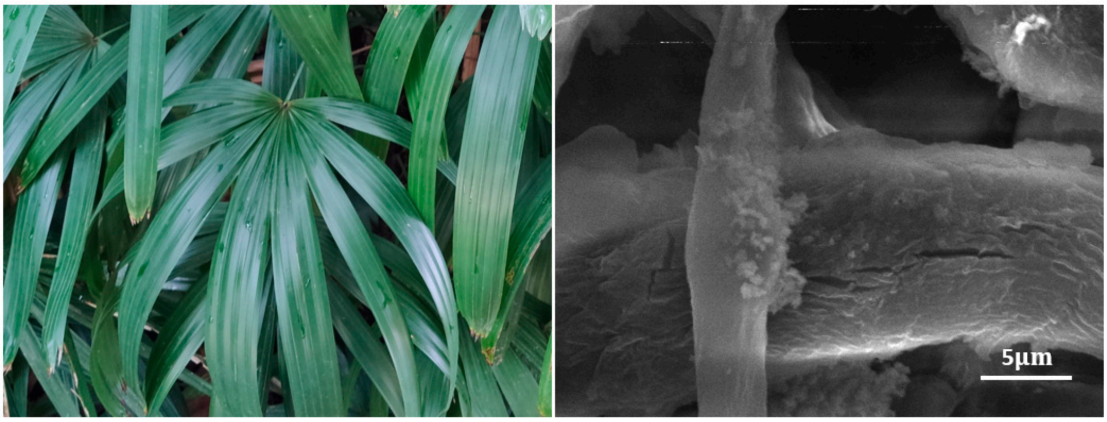

Supplementary Figure S8 The appearance and the SEM image of bamboo palm leaf in 5μm

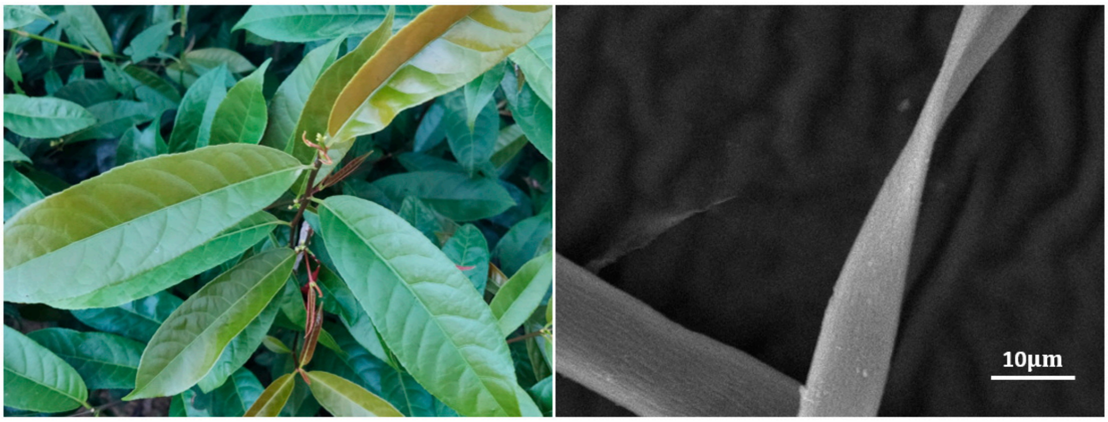

Supplementary Figure S9 The appearance and the SEM image of *Fissistigma oldhamii* leaf in 10μm

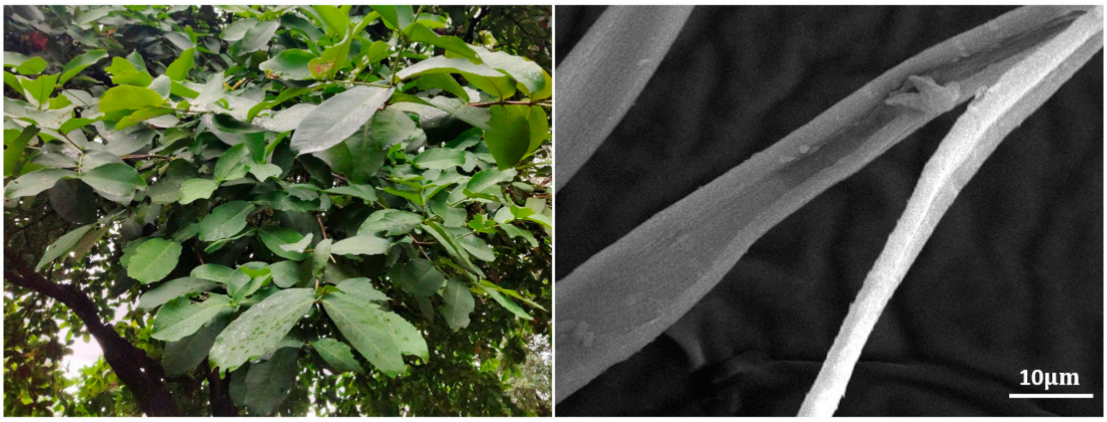

Supplementary Figure S10 The appearance and the SEM image of Semarang rose-apple leaf in 10μm

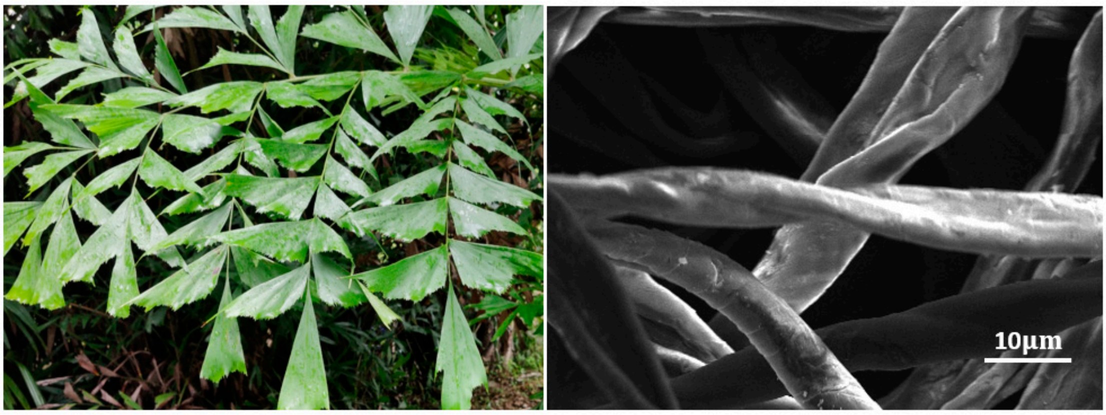

Supplementary Figure S11 The appearance and the SEM image of fishtail palm leaf in 10μm

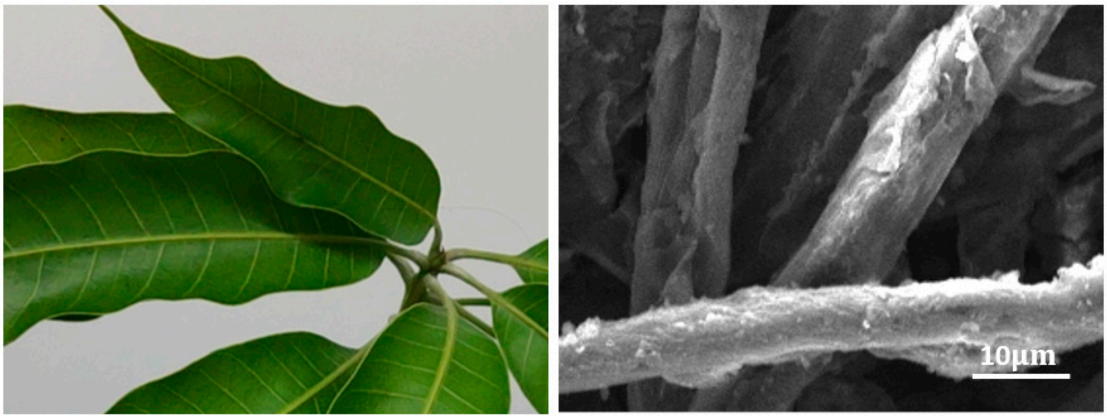

Supplementary Figure S12 The appearance and the SEM image of mango leaf in 10μm

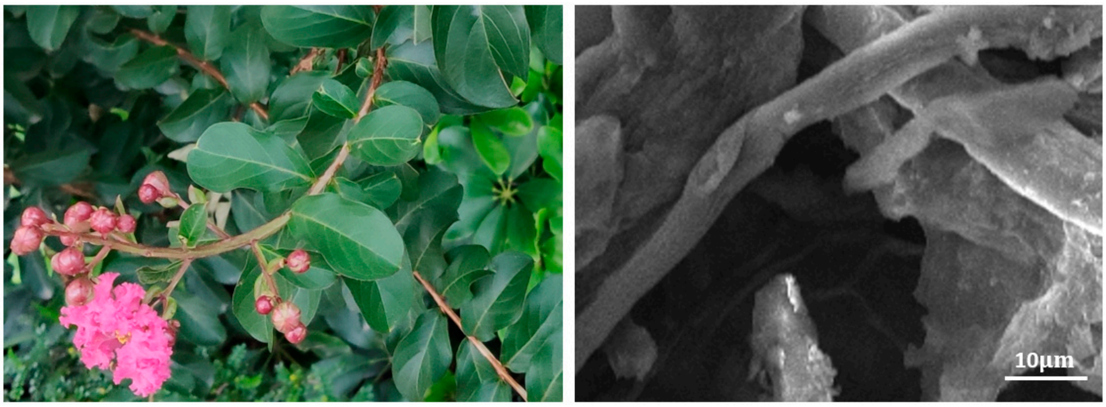

Supplementary Figure S13 The appearance and the SEM image of Lagerstroemia indica leaf in 10μm

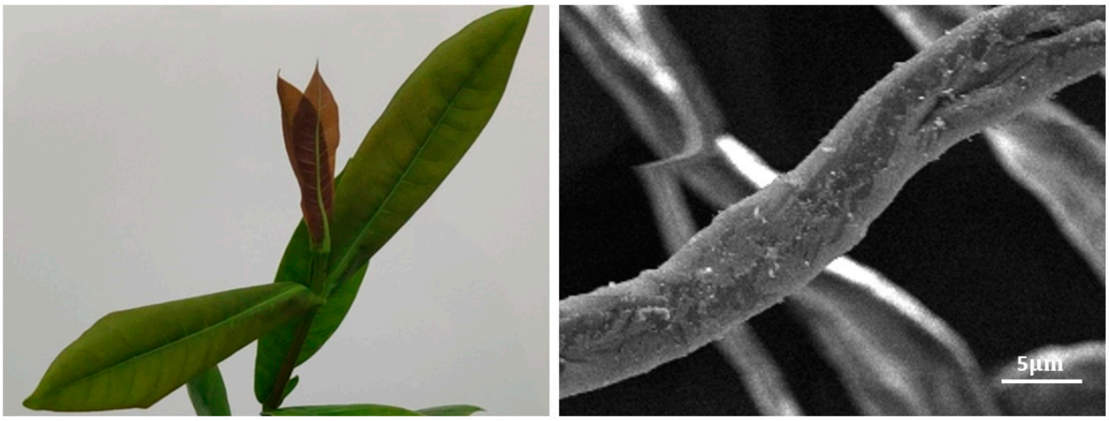

Supplementary Figure S14 The appearance and the SEM image of Chinese Ixora leaf in 10μm

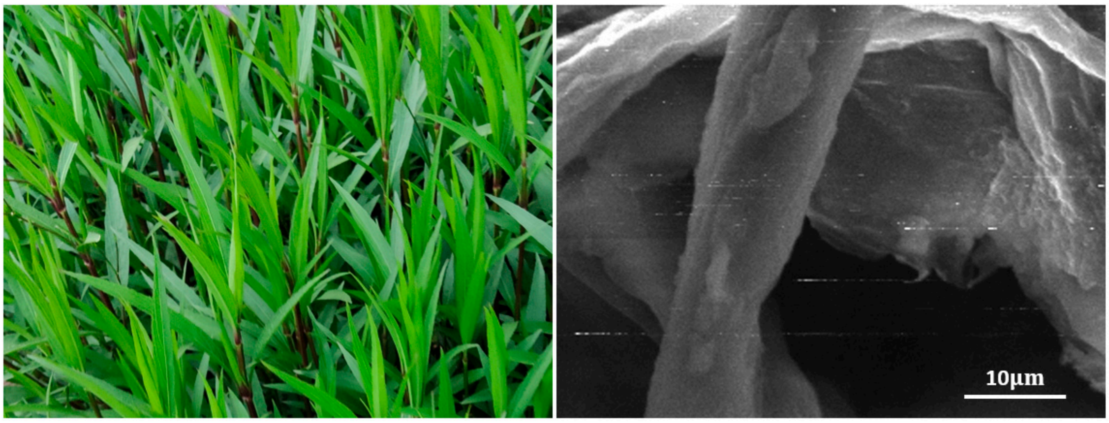

Supplementary Figure S15 The appearance and the SEM image of Britton's wild petunia leaf in 10μm

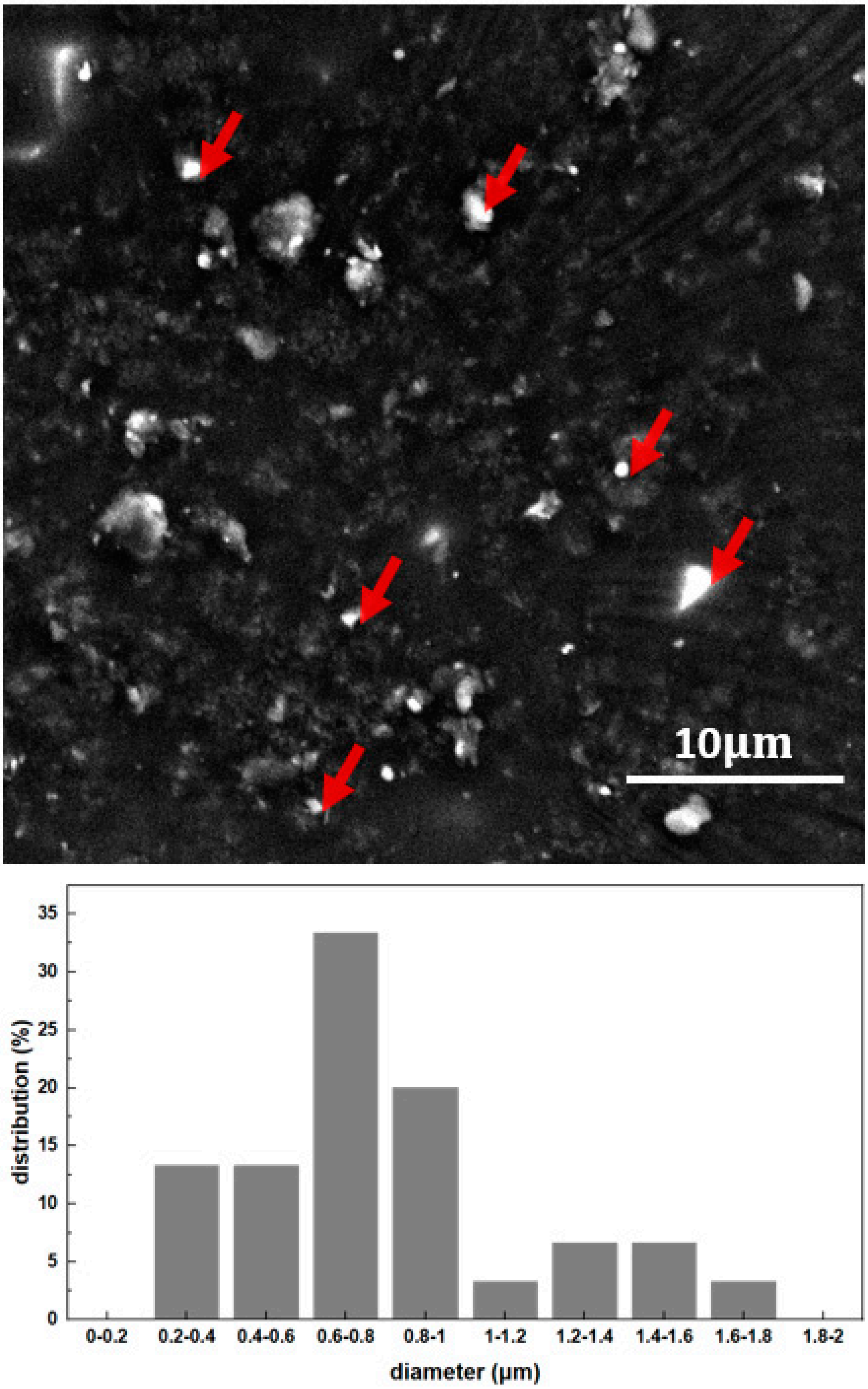

Supplementary Figure S16 The SEM image and size distribution of microplastic in the soil after degradation of the medical-surgical mask.

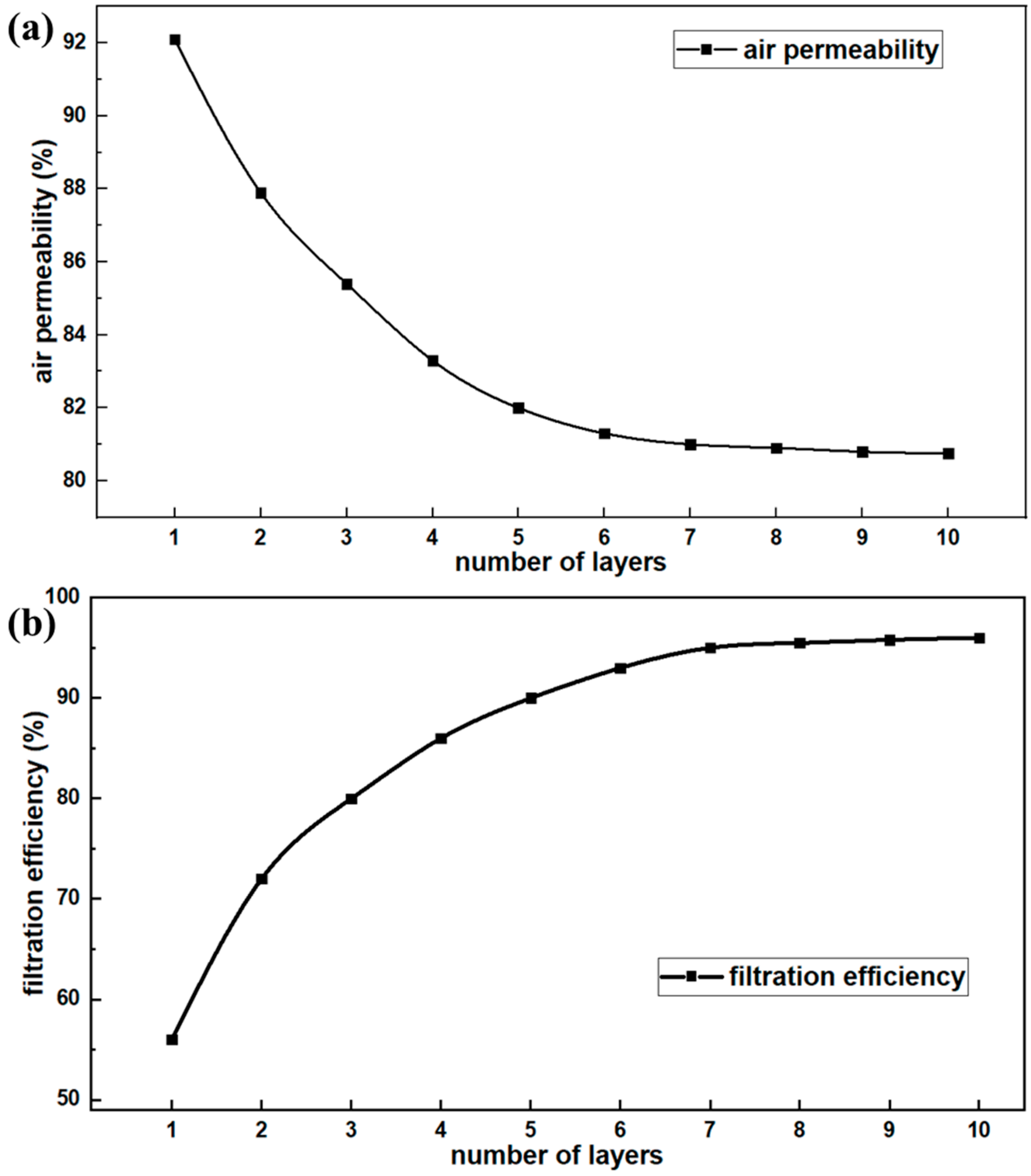

Supplementary Figure S17 The relationship between **(a)** the number of 3D printing layers and air permeability **(b)** the number of 3D printing layers and filtration efficiency of the TRespirator.

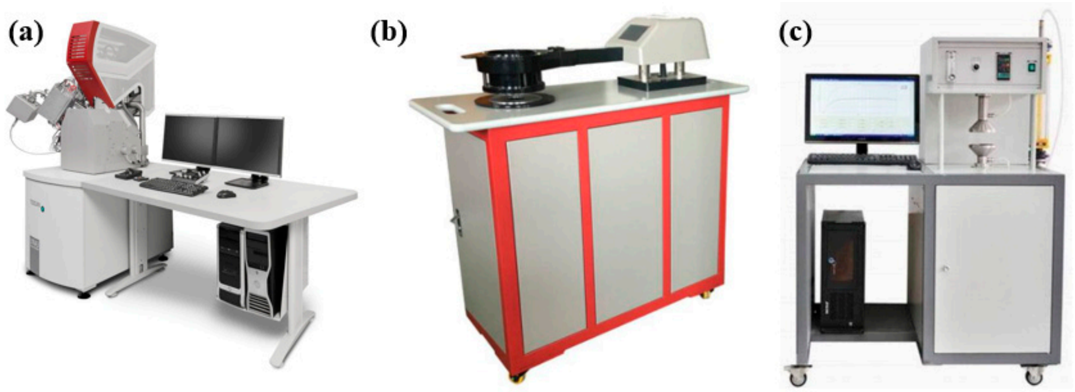

Supplementary Figure S18 Characterization and performance test machines (a) the SEM machine in the model MAIA3-TESCAN to characterize the mask samples and the leaves samples, **(b)** The air permeability testing machine in the model SG461-III from Changzhou Shuanggu Dunda Mechanical & Electrical Technology Co., Ltd.[40], **(c)** The PM removal machine from Germany Eagle Technology Inc.[41], which is used to test the PM removal performance of different masks.

Supplementary Table

Table S1 The cost for an N95 mask

content	Material name	Amount of consumable (gram per mask)	Unit price (Dollar per ton)	Cost (dollar per mask)
Raw material cost	PP non-woven cloth	1	1800	0.0018
	Melt-blown filter cloth	0.5	4100	0.00205
	Rubber band	0.2	1900	0.00038
	Aluminum plastic strip	0.3	3500	0.00105
	Packing bag	1.8	1100	0.00193
Processing cost				0.0008
Disinfection and quarantine cost				0.001
Production profit cost				0.002
Total cost				0.01101

Table S2 the diameter, pore size, with mean and maximum pore size respectively, and porosity of the non-woven medical surgical mask, hot air cotton layer of the KN95 mask, and hot air cotton layer of N95 mask.

Mask	Diameter (μm)	Pore size (μm)		Porosity (%)
		mean	maximum	
**Medical surgical mask**	19.6	42.72	129.4	81
**KN95 mask**	22.0	144.2	237.6	86
**N95 mask**	21.5	138.4	267.8	83

Table S3 the fiber diameter, fiber length, and porosity of 21 kinds of plants.

Plants	Fiber Diameter (μm)	Fiber length (μm)	Porosity (%)
Musa basjoo **leaf**	15.1	76.5	79.4
Ceiba speciosa **fiber**	15.7	87.6	81.1
Bauhinia purpurea **leaf**	12.0	42.3	56.2
Echinochloa crus-galli **grass**	4.9	90.8	54.4
Rhapis excelsa **leaf**	12.4	61.5	66.7
Bauhinia variegata **leaf**	7.2	51.6	63.6
Lagerstroemia indica **leaf**	8.9	54.3	64.2
Syzygium samarangense **leaf**	10.3	68.1	67.0
Carambola **leaf**	8.1	61.7	60.1
Hymenocallis littoralis **leaf**	7.1	43.1	48.1
Nandina domestica **leaf**	7.7	45.0	46.3
Mangifera indica **leaf**	8.6	57.6	66.4
Ixora Chinensis **leaf**	7.3	54.4	76.4
Ruellia simplex **leaf**	5.8	73.9	65.7
Duranta erecta **fiber**	6,2	45.3	78.9

Schefflera arboricola **fiber**	8,5	53.6	70.4
Caryota **fiber**	9,9	68.5	76.2
Phoenix loureiroi Kunth **fiber**	11,3	87.9	69.3
Chuniophoenix nana Burret **leaf**	13.5	72.9	71.3
Bambusoideae **leaf**	10.2	64.5	59.7
Bougainvillea **leaf**	7.1	63.1	65.3

Supplementary Video S1: 3D-printing process on the mixed photoresist
